# Adequacy of family history taking in ovarian cancer patients: a population-based study

**DOI:** 10.1007/s10689-012-9518-6

**Published:** 2012-03-03

**Authors:** Anne M. van Altena, Sandra van Aarle, Lambertus A. L. M. Kiemeney, Nicoline Hoogerbrugge, Leon F. A. G. Massuger, Joanne A. de Hullu

**Affiliations:** 1 Department of Obstetrics and Gynecology, Radboud University Medical Centre, P.O. Box 9101, 6500 HB Nijmegen, The Netherlands; 2 Department of Epidemiology, Biostatistics and Health Technology Assessment, Radboud University Medical Centre, Nijmegen, The Netherlands; 3 Department of Urology, Radboud University Medical Centre, Nijmegen, The Netherlands; 4 Comprehensive Cancer Center The Netherlands, Utrecht, The Netherlands; 5 Department of Human Genetics, Radboud University Medical Centre, Nijmegen, The Netherlands

**Keywords:** Family history taking, Ovarian neoplasms, Hereditary neoplastic syndromes

## Abstract

The aim of this study was to evaluate the adequacy of family history taking in epithelial ovarian cancer (EOC) patients and to identify factors that determine adequacy. Furthermore, the validity of family history taking was assessed by comparison with self-administered questionnaires. Medical records of all 1,112 EOC patients registered by the nation-wide cancer registry and diagnosed in eleven Dutch hospitals between 1996 and 2006 were reviewed. Adequate family history taking was defined as a written notification of the presence or absence of relatives with breast or ovarian cancer. Factors that were correlated with family history taking were identified using univariable and multivariable logistic regression. 147 patients filled in a postal questionnaire. An adequate family history was taken in 41% of all cases. Younger age, an academic hospital and having undergone surgery and/or chemotherapy were associated with adequate family history taking. The comparison with self-administered questionnaires showed a disagreement in 64% mainly due to missing data in medical records. Documentation on family history is either absent or inadequate in the medical records in the majority of EOC patients. These data urge for better uptake of hereditary cancer risk assessment. Different strategies for this assessment like improved family history taking and genetic testing in EOC patients should be explored.

## Introduction

Although many theories have been proposed, the mechanism of ovarian carcinogenesis is still unclarified. Several risk factors for epithelial ovarian cancer (EOC) are identified. However, by far the most significant risk factor for EOC is a family history of this disease; a woman with one first-degree relative with ovarian cancer has a three-fold increased risk of developing EOC herself [1]. It is estimated that approximately 10–15% of ovarian cancer is hereditary [2–5]. At least two hereditary syndromes predispose to familial ovarian cancer: the hereditary breast-ovarian cancer (HBOC) syndrome and the Lynch syndrome. Mutations in the *BRCA1* and *BRCA2* tumor suppressor genes account for 65–85% of all hereditary ovarian cancers. A recent population-based study showed a combined *BRCA1* and *BRCA2* mutation frequency of 13.3% among 1,342 women with ovarian cancer [6]. The lifetime risk of ovarian cancer in *BRCA1* and *BRCA2* mutation carriers is approximately 40–60 and 10–25%, respectively [7, 8]. Mutations in mismatch repair genes *MLH1, MSH2, MSH6, PMS1*, and *PMS2* in the Lynch syndrome account for 10–15% of hereditary ovarian cancers, with a lifetime risk of 8–10% of developing ovarian cancer [2, 8, 9].

To date, reliable screening methods for ovarian cancer are not available and screening for ovarian cancer in the general population does not reduce mortality [10]. Even in high risk populations screening has a poor ability to detect early stage disease [11]. The only proven method to dramatically reduce the incidence of ovarian cancer in high-risk patients is a prophylactic bilateral salpingo-oophorectomy (BSO) [12, 13]. BSO is indicated in *BRCA*-carriers around the age of 40. It not only reduces the risk of ovarian cancer by up to 96% but also halves the risk of breast cancer (BC) in pre-menopausal women [12–15].

The primary tool to trace hereditary cancer is family history taking. It has several advantages over genetic tests including lower costs, greater acceptability and a reflection of shared genetic and environmental factors [16]. Moreover, criteria for genetic testing rely almost exclusively on family history information [17]. In various studies however, the question is raised how accurate family history is to predict mutation status [3, 4, 6]. Several reasons are given for a low validity of family history taking as diagnostic test for hereditary cancer, i.e. small families or families with few women, changeable penetrance, sporadic cancers, new mutations or inadequate family history taking. There is no literature available on the adequacy of family history taking in EOC patients. Studies addressing this issue have mainly focused on colorectal and BC.

The purpose of this population-based study was to describe adequacy of family history taking in EOC patients and to identify factors that determine this adequacy. Secondly, we aimed to acquire insight in the reliability of presence or absence of written notifications of family history in medical records by comparing data in medical records with data collected thru self-administered questionnaires.

## Patients and methods

To evaluate adequacy of family history taking in EOC patients and factors that determine this adequacy, we used population-based data from a retrospective study in 11 hospitals in the eastern part of the Netherlands: one university clinic and ten community hospitals. The population-based Netherlands Cancer Registry (NCR) registered 1,178 patients with primary EOC in these hospitals between 1996 and 2006. Hospital records of the patients were studied by trained registrars using a standard case record form. Data on patient characteristics, tumor characteristics, therapy and recurrence were collected. After excluding 66 patients without histological confirmed EOC, data of a total number of 1,112 patients were available. Data on family history taking included degree and age at diagnosis of all relatives diagnosed with EOC, BC, or colorectal cancer (CRC). For this study adequate family history taking was defined as a written notification of the presence or absence of relatives with breast or ovarian cancer. Factors that may be correlated with family history taking were tested using univariable logistic regression analysis. Factors tested were; age of the patient at diagnosis (<40, 40–60, >60 year), hospital type (general, teaching, university hospital), year of diagnosis (1996–1999, 2000–2003, 2004–2006), menstrual state (pre- or postmenopausal), cancer in patient’s history (yes or no), BC in patient’s history (yes or no), cervical cancer in patient’s history (yes or no), endometrial cancer in patient’s history (yes or no), colon cancer in patient’s history (yes or no), CA125 at diagnosis (≤35 or >35), Risk of Malignancy Index (≤200 or >200), Karnofsky score (≤70 or >70), stage of disease (early = FIGO < IIb or advanced = FIGO ≥ IIb), surgery (yes or no), chemotherapy (yes or no), histology (serous, endometrioid, mucinous, adenocarcinoma not otherwise specified or other), grade of differentiation (grade I or II or III), number of recurrences (0, 1, 2, 3 or ≥4) and inclusion in a trial (yes or no). All *P* values presented are two-sided, and associations were considered significant if the *P* value <0.05. Since correlation between certain factors was expected all significant indicators (*P* < 0.05) were entered in a multivariable model using a stepwise forward approach. Statistical analyses were performed using Statistical Package for Social Sciences 16.0 for Microsoft Windows (SPSS Inc.).

**Fig. 1:**
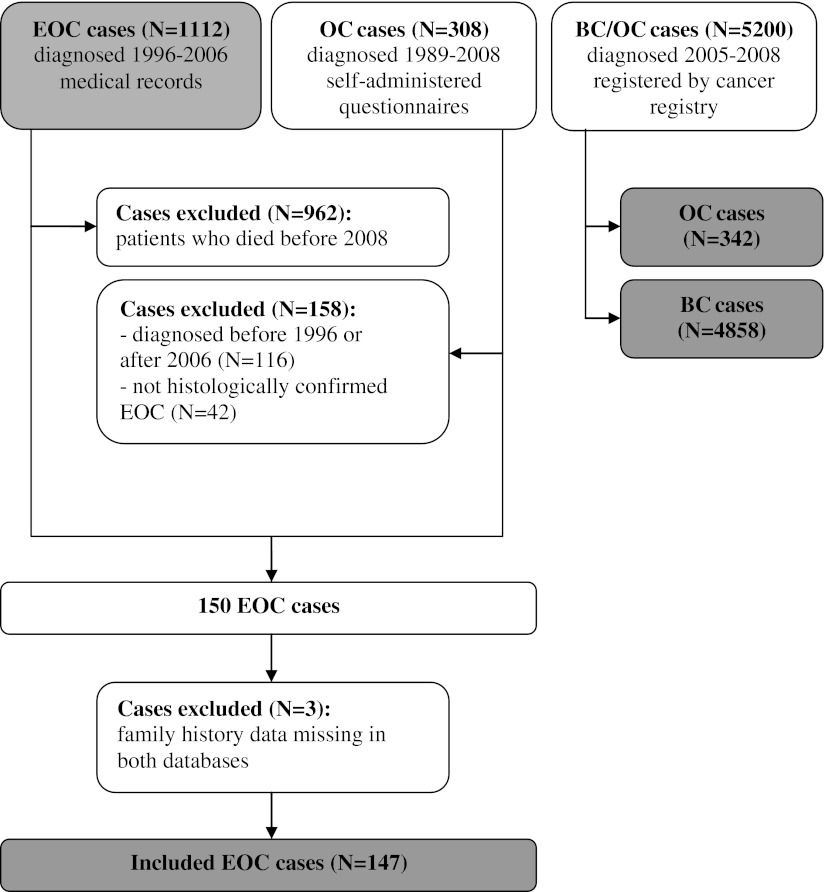
Overview of databases used in this study. *EOC* epithelial ovarian cancer, *OC* ovarian cancer, *BC* breast cancer

To measure the reliability of presence or absence of written notifications on family history in medical records, we compared data in the medical files with data collected by self-administered questionnaires. In 2008, these questionnaires were sent to all living patients diagnosed with ovarian cancer between 1989 and 2008 in seven of the 11 hospitals. The questionnaire database included 308 patients of whom 150 were also included in the above described EOC database. The other 158 cases were diagnosed before 1996 or after 2006, or were non-EOC cases (Fig. 1). Another three cases were excluded since data on family history were missing in both the EOC database and the questionnaires. Data on family history taking included type of malignancy and age at diagnosis of first-degree relatives. A comparison was made based on the number of relatives with a malignancy, the type of malignancy (being BC, EOC or CRC), and age at diagnosis. Cancer cases among family members mentioned in the self-questionnaire but diagnosed after the last follow-up date of the patient were excluded. Agreement between the databases on number of relatives with a malignancy, type of malignancy, and age at diagnosis with an acceptable margin of error of 5 years, was defined as total agreement. Partial agreement was defined as agreement on number of relatives with a malignancy and type of malignancy.

## Results

In total 1,112 medical records were studied. Figure 1 shows an overview of this database and the other databases used in this study. For 41% (456/1,112) of the cases, documentation on family history of breast and ovarian cancer was found in the medical records. Table 1 shows characteristics of patients with or without an adequate documentation on family history. Univariable logistic regression analysis showed age, hospital type, year of diagnosis, menstrual state, BC in the past, Karnofsky score, histology, having surgery or chemotherapy, recurrence, and inclusion in a trial, to be significantly correlated with adequacy of family history taking. Multivariable logistic regression analysis identified age, hospital type, and having surgery or chemotherapy, as significant independent prognostic factors (Table 2). Patients who were younger, diagnosed in a university hospital, or who underwent chemotherapy and/or surgery, were more likely to have an adequate documentation on family history.

**Table 1:** Associations between patient, tumor and treatment characteristics and adequacy of family history taking in univariable logistic regression analysis (n = 1,112)

Characteristics	Adequate N(%)	Inadequate N(%)	*P* value
Age (years)			<0.01
>60	200 (32%)	429 (68%)	
40–60	216 (52%)	202 (48%)	
<40	39 (66%)	20 (34%)	
Hospital type			<0.01
General hospital	182 (33%)	371 (67%)	
Teaching hospital	188 (42%)	264 (58%)	
University hospital	86 (81%)	20 (19%)	
Year of diagnosis			0.019
1996–1999	166 (38%)	274 (62%)	
2000–2003	181 (41%)	265 (59%)	
2004–2006	109 (49%)	113 (51%)	
Menstrual state			<0.01
Premenopausal	113 (57%)	86 (43%)	
Postmenopausal	311 (37%)	527 (63%)	
Cancer in patients history			0.230
No	372 (41%)	536 (59%)	
Yes	82 (46%)	97 (54%)	
Breast cancer in patients history			<0.01
No	410 (41%)	599 (59%)	
Yes	44 (56%)	34 (44%)	
Colon cancer in patients history			0.464
No	448 (42%)	621 (58%)	
Yes	6 (33%)	12 (67%)	
Endometrial cancer in history			0.131
No	443 (42%)	607 (58%)	
Yes	11 (30%)	26 (70%)	
Cervical cancer in patients history			0.593
No	448 (42%)	628 (58%)	
Yes	5 (50%)	5 (50%)	
CA-125 at diagnosis			0.174
≤35	60 (49%)	63 (51%)	
>35	360 (42%)	491 (58%)	
Risk of malignancy index (RMI)			0.984
≤200	56 (44%)	71 (56%)	
>200	286 (44%)	364 (56%)	
Karnofsky score			<0.01
<70	51 (24%)	159 (76%)	
≥70	395 (46%)	459 (54%)	
Stage			0.602
Early (<IIb)	113 (43%)	152 (57%)	
Advanced (≥IIb)	316 (44%)	394 (56%)	
Chemotherapy			<0.01
No	98 (25%)	297 (75%)	
Yes	357 (50%)	359 (50%)	
Surgery			<0.01
No	40 (19%)	166 (81%)	
Yes	416 (46%)	488 (54%)	
Histology			<0.01
Serous	205 (48%)	221 (52%)	
Mucinous	37 (42%)	52 (58%)	
Endometrioid	78 (47%)	89 (53%)	
Adenocarcinoma NOS^a^	84 (33%)	169 (67%)	
Other^b^	45 (40%)	67 (60%)	
Grade of differentiation			0.297
1	56 (41%)	81 (59%)	
2	100 (42%)	140 (58%)	
3	225 (47%)	257 (53%)	
Number of recurrence			<0.01
0	229 (35%)	426 (65%)	
1	145 (47%)	163 (53%)	
2	58 (53%)	51 (47%)	
3	14 (61%)	9 (39%)	
≥4	10 (59%)	7 (41%)	
Inclusion in trial			0.034
No	421 (40%)	623 (60%)	
Yes	33 (54%)	28 (46%)	

Adequate family history taking was defined as a written notification of the presence or absence of relatives with breast or ovarian cancer

^a^Adenocarcinoma not otherwise specified

^b^Clear cell, Brenner, mixed, undifferentiated and other rare epithelial types

**Table 2:** Associations between patient, tumor and treatment characteristics and adequacy of family history taking in multivariable logistic regression analysis (n = 1,112)

Characteristics	Adequate	Inadequate	Odds ratio	95% CI	
				Lower	Upper
Age					
>60	200 (32%)	429 (68%)	1.00		
40–60	216 (52%)	202 (48%)	1.65	1.23	2.22
<40	39 (66%)	20 (34%)	3.27	1.70	6.26
Hospital type					
General hospital	182 (33%)	371 (67%)	1.00		
Teaching hospital	188 (42%)	264 (58%)	1.63	1.22	2.19
University hospital	86 (81%)	20 (19%)	8.74	4.84	15.79
Surgery					
No	40 (19%)	166 (81%)	1.00		
Yes	416 (46%)	488 (54%)	1.68	1.02	2.76
Chemotherapy					
No	98 (25%)	297 (75%)	1.00		
Yes	357 (50%)	359 (50%)	2.25	1.63	3.12

Adequate family history taking was defined as a written notification of the presence or absence of relatives with breast or ovarian cancer

147 cases in the EOC database also completed a questionnaire. In 36% (53/147) of the cases, full agreement was found regarding the number and types of malignancy, as well as ages at diagnoses within 5 years. Another five per cent (8/147) agreement was found for the number and type of malignancy in relatives. In 59% (86/147) of the cases, the information on family cancer history was discordant. In those 86 cases, medical records reported more malignancies than self-administered questionnaires in 11% (9/86), self-administered questionnaires reported more malignancies in 23% (20/86), and data were missing in the medical records where the self-administered questionnaires reported a negative family history in 66% (57/86).

## Discussion

Our population-based study shows that, adequate documentation on family history was present in only 41% of all medical records of EOC patients. This percentage is in agreement with ranges found in literature regarding patients with CRC and data required in primary care settings [17–20]. In univariable analysis a slight improvement over time was seen which is encouraging however family history is still poorly recorded. This is especially true when taking into account our limited definition of an adequate history covering the majority of hereditary ovarian cancers, namely the *BRCA* mutation carriers, but leaving others unattended. Previous studies on CRC defined adequate family history taking by presence of a notification of relatives with CRC only [18, 20]. Therefore, we feel that we used an appropriate definition for adequate family history taking in this study, but encourage clinicians to apply a much broader definition.

We believe a comprehensive adequate family history should include information on first and second degree relatives, the type of tumor they developed (especially colorectal or endometrial carcinoma) and the age at onset. Since families are getting smaller one should also note the total number of first and second-degree relatives to put it in perspective. An additional problem is that with the decreasing number of relatives family history taking is expected to be less accurate in the future. It is important to ask about second degree relatives since 50% of the mutations are paternally derived and the fathers are likely to be unaffected. Though it is known that information on second degree relatives is less reliable than information on first degree relatives [21]. Also the absence of malignancies should be noted. In case of suspicion of hereditary cancer one should refer the patient to a specialist in the field of clinical genetics.

We defined four factors to be independently correlated with adequacy of family history taking. Having chemotherapy or surgery often requires involvement of more specialists, longer therapeutic relationships and hospitalization, leading to more opportunities to ask about family history. Regarding age, in younger patients family history taking is probably more accurate because physicians are aware of an earlier age at onset in the majority of mutation carriers. We urge specialists to keep in mind the possibility of a hereditary malignancy, even in the elderly patients. With respect to hospital type, specialized gynecologists in academic hospitals more often recorded family history. Since it is shown that treatment by a gynecologic oncologist improves outcome, more and more EOC patients will be treated by specialized gynecologists and discussed in multidisciplinary tumor boards, which eventually may improve adequacy of history taking.

With the result of the multivariable analysis the question raised if gynecologists perform worse in family history taking compared to other physicians. To answer this question we again used the database of the NCR. Family history data of all 4,858 BC and all 342 EOC patients diagnosed between 2005 and 2008 were compared. Data, extracted from medical records, included number of first-degree relatives with the same malignancy (being BC or EOC), age of the youngest relative with the same malignancy and number of first-degree relatives with another malignancy. In 30% (104/342) of all EOC patients, compared with 64% (3,103/4,858) of all BC patients, documentation on presence or absence of first-degree relatives with the same malignancy was found. 24% (81/342) of EOC patients and 20% (989/4,858) of BC patients had any documentation in their medical record about first-degree relatives with other malignancies. So family history regarding first-degree relatives with the same malignancy was taken twice more frequently in BC patients. Breast cancer is much more common compared to ovarian cancer. As a result, patients can be more aware of BC in their family and both patient and physician can be more forthcoming towards this subject. The clinician and patient can also be more aware of BC as a hereditary cancer, since there has been a lot of attention for this subject. Family history taking on first-degree relatives with another malignancy is as likely to be forgotten by surgeons in BC patients as by gynecologists in EOC patients.

Although the large sample size is an apparent strength of this study, we are aware of some limitations. It is possible that specialists did not register anything in the absence of relatives with a malignancy. In that case the family history was considered to be absent although it in fact was examined correctly. In order to determine this possible bias, we compared the data with self-administered questionnaires. Ziogas et al. [21] showed that reliability of self-reported family history taking varies by cancer site and by degree of relative. For first-degree relatives, family history provided by patients was accurate in 83.3% for ovarian cancer. Murff et al. [22] supported the first statement but also states that negative family history reports for ovarian cancer are less useful. Especially abdominal malignancies are reported inaccurately probably because many organs are within the abdominal cavity and it is often referred to as “abdominal cancer” [23]. In the current study, we were unable to assess the reliability of family history taking by verifying it with a population-based registration and therefore chose a comparison with self-administered questionnaires.

Data of 147 EOC cases of mainly long-term ovarian cancer survivors were studied. In nearly 60% of the cases no agreement was found which was largely due to missing data in the EOC database in absence of any relatives with a malignancy in the questionnaires. Data from medical records were gathered in 2007 and the questionnaires were filled out by patients in 2008. It is likely that if gynecologists asked about family history, they did so in the early stages of treatment and never pursued it over time. Patients may also have developed more awareness on cancer in their family during the years since their diagnoses. As mentioned previously, these 147 cases were mainly long-term survivors. While analyzing data of these cases, 58% of these patients showed an adequate documentation in their medical records, compared to 41% in the whole EOC group. An unselected patient group with respect to survival may worsen the results in our study even more.

It is of upmost importance to have an accurate tool for the identification of hereditary cancer. The risk of EOC can currently not be reduced by screening but a prophylactic BSO offered to high-risk patients can reduce the risk significantly. BRCA mutation carriers also have an improved sensitivity to platinum chemotherapy and novel therapeutic agents such as poly (ADP-ribose) polymerase inhibitors have increased activity in these patients [24]. High risk patients however, need to be identified firstly. Our study shows that family history taking over the last 10 years was inadequate in the majority of EOC patients. Moreover, a recent study shows that family histories change significantly over time and updates on family history every 5–10 years are recommended [25]. Data on sensitivity of family history as a predictor of mutation carrier status are conflicting. In various studies all EOC patients both underwent family history taking and genetic testing. The proportion of BRCA gene mutation carriers having a first or second degree relative with ovarian or BC varied from 92% [26] to 62–69% [4, 5]. It appears reasonable to offer genetic testing to all non-mucinous EOC patients in order to fully benefit from preventive measures like a BSO. But since genetic testing is not routinely offered in many countries and even when it is offered it is not performed in all cases, there is still a place for family history taking. Family history taking also plays an important role in the counseling of patients with a possible hereditary tumor. Moreover, patients with familial ovarian cancer but without mutation can be offered a BSO. The upcoming of electronic health record systems can be helpful by turning family history taking into a fixed item in consultations with cancer patients. Further education is needed for the physician to increase awareness of hereditary cancer since taking an adequate family history is still essential to provide high-standard care to patients with cancer and their families even in the era of genetic testing.
